# Light Control of Salt-Induced Proline Accumulation Is Mediated by ELONGATED HYPOCOTYL 5 in *Arabidopsis*

**DOI:** 10.3389/fpls.2019.01584

**Published:** 2019-12-10

**Authors:** Hajnalka Kovács, Dávid Aleksza, Abu Imran Baba, Anita Hajdu, Anna Mária Király, Laura Zsigmond, Szilvia Z. Tóth, László Kozma-Bognár, László Szabados

**Affiliations:** ^1^Institute of Plant Biology, Biological Research Centre, Szeged, Hungary; ^2^Department of Genetics, Faculty of Sciences and Informatics, University of Szeged, Szeged, Hungary

**Keywords:** ELONGATED HYPOCOTYL 5, proline accumulation, Arabidopsis, light signalling, gene expression regulation

## Abstract

Plants have to adapt their metabolism to constantly changing environmental conditions, among which the availability of light and water is crucial in determining growth and development. Proline accumulation is one of the sensitive metabolic responses to extreme conditions; it is triggered by salinity or drought and is regulated by light. Here we show that red and blue but not far-red light is essential for salt-induced proline accumulation, upregulation of *Δ1-PYRROLINE-5-CARBOXYLATE SYNTHASE 1* (*P5CS1*) and downregulation of *PROLINE DEHYDROGENASE 1* (*PDH1*) genes, which control proline biosynthetic and catabolic pathways, respectively. Chromatin immunoprecipitation and electrophoretic mobility shift assays demonstrated that the transcription factor ELONGATED HYPOCOTYL 5 (HY5) binds to G-box and C-box elements of *P5CS1* and a C-box motif of *PDH1*. Salt-induced proline accumulation and *P5CS1* expression were reduced in the *hy5hyh* double mutant, suggesting that HY5 promotes proline biosynthesis through connecting light and stress signals. Our results improve our understanding on interactions between stress and light signals, confirming HY5 as a key regulator in proline metabolism.

## Introduction

Proline accumulates to high levels in plants at low water potential caused by drought, salinity and in response to several other abiotic and biotic stresses (Kemble and Macpherson, 1954; Schat et al., 1997; Fabro et al., 2004; Yang et al., 2009; Szabados and Savoure, 2010; Aleksza et al., 2017). Proline was suggested to act as osmoprotectant stabilizing enzymes or maintaining redox equilibrium in adverse conditions (Delauney and Verma, 1993; Székely et al., 2008; Szabados and Savoure, 2010; Verslues and Sharma, 2010; Kavi Kishor and Sreenivasulu, 2014; Per et al., 2017). Free proline content is defined by biosynthesis and degradation, and modulated by transport, protein synthesis, and degradation (Lehmann et al., 2010; Verslues and Sharma, 2010; Hildebrandt, 2018). The main biosynthetic pathway has two consecutive steps catalyzed by the rate-limiting *Δ* *^1^* *-pyrroline-carboxylate synthetase* (*P5CS*) enzyme (Hu et al., 1992) followed by P5C reductase (P5CR) (Delauney and Verma, 1990). Proline biosynthesis takes place in cytosol, although localization of P5CS1-GFP protein in chloroplasts of salt-treated cells suggest that biosynthesis may take place in plastids under stress conditions (Székely et al., 2008). Proline degradation is a mitochondrial oxidative process, mediated by the rate-limiting proline dehydrogenase (PDH) and P5C dehydrogenase (P5CDH) enzymes (Kiyosue et al., 1996; Servet et al., 2012). In most plants P5CS is encoded by two genes. In *Arabidopsis P5CS1* (*AT2G39800*) responds to hyperosmotic stress and is regulated by ABA-dependent and independent signals, whereas *P5CS2* (*AT3G55610*) is considered to be a housekeeping gene, which can be induced by certain pathogens *via* salicylic acid-dependent signals (Savouré et al., 1997; Strizhov et al., 1997; Fabro et al., 2004; Székely et al., 2008; Sharma and Verslues, 2010). *PDH1* (*AT3G30775*) is repressed in high osmotic conditions and is induced by proline during stress recovery (Kiyosue et al., 1996). Compared to *PDH1*, *PDH2* has a very low expression level, which is however induced during phosphate starvation (Aleksza et al., 2017). ABA, reactive oxygen species, calcium, and lipid signals were implicated in the regulation of proline metabolism (Thiery et al., 2004; Parre et al., 2007; Ben Rejeb et al., 2015). Although important progress has been made in the last few years, transcription regulation of key genes in proline metabolism is far from well understood. A number of *cis* regulatory sequences have been identified in promoters or 5′UTRs of key metabolic genes, but direct evidence on promoter-binding transcription factors and their function is scarce (Fichman et al., 2015; Zarattini and Forlani, 2017). We have recently described that PHOSPHATE STARVATION RESPONSE 1 (PHR1) and PHOSPHATE STARVATION RESPONSE LIKE-1 (PHL1) transcription factors bind the P1BS motif in the first intron of *P5CS1*, upregulate its expression, and promote proline accumulation during phosphate starvation (Aleksza et al., 2017). A recent report showed that the transcription factor ANAC55 (*Arabidopsis* NAM, ATAF, and CUC) is a positive regulator of *P5CS1* expression and proline accumulation in high osmotic conditions, although direct binding to *P5CS1* promoter elements was not demonstrated (Fu et al., 2018). Allelic variation in the barley *P5CS1* gene was recently reported, showing that promoter mutations in the abscisic acid-responsive element (ABRE) can considerably alter *P5CS1* expression, proline accumulation, and drought tolerance (Muzammil et al., 2018). Some information is available on transcriptional regulation of *PDH1*. Basic leucine zipper (bZIP) transcription factors of the ATB2 subgroup were shown to upregulate *PDH1* expression during hypoosmolarity through binding to the ACTCAT *cis*-acting promoter element (Satoh et al., 2002; Satoh et al., 2004; Weltmeier et al., 2006). Chromatin immunoprecipitation (ChIP) analysis confirmed that bZIP1 and bZIP53 factors bind to the *PDH1* promoter and upregulate it in low energy conditions (Dietrich et al., 2011). Besides transcriptional control, epigenetic regulation and alternative splicing were shown to influence the expression of the *P5CS1* and *PDH1* genes (Kesari et al., 2012; Jimenez-Arias et al., 2015). Histone methylation was recently shown to control stress memory response of *P5CS1* in *Arabidopsis* (Feng et al., 2016).

Light was found to influence proline levels by inducing *P5CS1* and repressing *PDH1* expression (Hayashi et al., 2000; Abraham et al., 2003; Diaz et al., 2005). While considered as a housekeeping gene, *P5CS2* was identified as a target of CONSTANS (CO) and is therefore also subject to light and flowering time regulation (Samach et al., 2000). Datamining of publicly available transcript profiling data (Dubois et al., 2017) suggested reciprocal fluctuation of the expression of the *P5CS1* and *PDH1* genes in response to light and drought (**Figure S1**).

Light can influence gene expression in various ways. Light perception through photoreceptors is mediated by phytochromes (PHYA-E) absorbing red/far-red light, cryptochromes (CRY1-2) sensing blue light and phototropins (PHOT1-2), which absorb blue and additionally UV-A light (Briggs and Christie, 2002; Franklin and Quail, 2010; Kami et al., 2010; Chaves et al., 2011). bHLH-type phytochrome-interacting factors repress photomorphogenic development and promote the expression of light-repressed genes, but are degraded upon interaction with the active forms of phytochrome receptors (Leivar and Monte, 2014). The bZIP-type transcription factor ELONGATED HYPOCOTYL 5 (HY5) is a phytochrome-interacting factor antagonist that acts downstream of virtually all classes of photoreceptors, and promotes photomorphogenesis and the expression of light-induced genes (Cluis et al., 2004; Toledo-Ortiz et al., 2014). Crosstalk between light and several other signaling pathways has been demonstrated, in which HY5 can function as a signaling hub. HY5 directly interacts with ACGT-containing (ACE) Light-Responsive Elements in the promoters of light-induced genes and upregulates their transcription (Chattopadhyay et al., 1998). Signals from photoreceptors promote accumulation of HY5 at transcriptional and posttranscriptional levels (Binkert et al., 2014; Sheerin et al., 2015), but apparently do not affect the DNA-binding affinity or specificity of the HY5 protein (Hajdu et al., 2018). HY5 lacks any domains with transcriptional regulator function, thus it requires co-factors to control gene expression (Li et al., 2010) and is supposed to act as a component of multiprotein complexes. More recently the role of HY5 in multiple signaling systems was uncovered, showing that, together with the closely related HY5-HOMOLOG (HYH) factor, it integrates light, hormonal, and developmental regulation through multiple interactions with other transcription factors and regulatory proteins (Gangappa and Botto, 2016). A recent paper described that the stress-induced transcription memory of *P5CS1* is influenced by light and is mediated by HY5, able to bind to C/A-box sequence elements in the *P5CS1* promoter and 5′ UTR region (Feng et al., 2016). HY5 was also shown to modulate ABA signaling by promoting *ABI5* expression through binding to its promoter (Chen et al., 2008). Light and ABA regulation is influenced by the C2H2-type zinc finger protein ZFP3, which represses ABA signals and promotes photomorphogenesis (Joseph et al., 2014). Responses to light signals can be fine-tuned by the EREBP-type ABI4 which is implicated in ABA and sugar signaling (Wind et al., 2012). Interacting light, ABA and stress signals are therefore influenced by different sets of transcription factors such as the bZIP-type HY5 and ABI5, the C2H2-type ZFP3, or the EREBP-type ABI4.

In addition to perception through photoreceptors, light also affects the expression of a set of nuclear genes by chloroplast retrograde signaling which depends on light reactions of photosynthesis (Gollan et al., 2015). In this regulatory system chloroplasts acts as sensors and signaling components include sugar and carotenoid metabolites, reactive oxygen species, plastoquinone pool redox state, and various classes of regulatory proteins such as protein kinases and transcription factors. Chloroplast-derived signals control chloroplast development and responses to environmetal stresses (Fey et al., 2005; Fernandez and Strand, 2008; Gollan et al., 2015; Kleine and Leister, 2016; D’Alessandro et al., 2018).

This communication focuses on the light-dependent control of proline metabolism. We show that HY5 binds to conserved sequence elements of the *P5CS1* and *PDH1* genes and can positively contribute to salt-induced proline accumulation. HY5 seems to function as a regulatory hub that integrates light and stress signals in the control of proline metabolism. We conclude that proline metabolism is controlled by multiple regulatory pathways and is influenced by interacting stress and light signals.

## Results

### Proline Accumulation Is Influenced by Light

**Figure 1:**
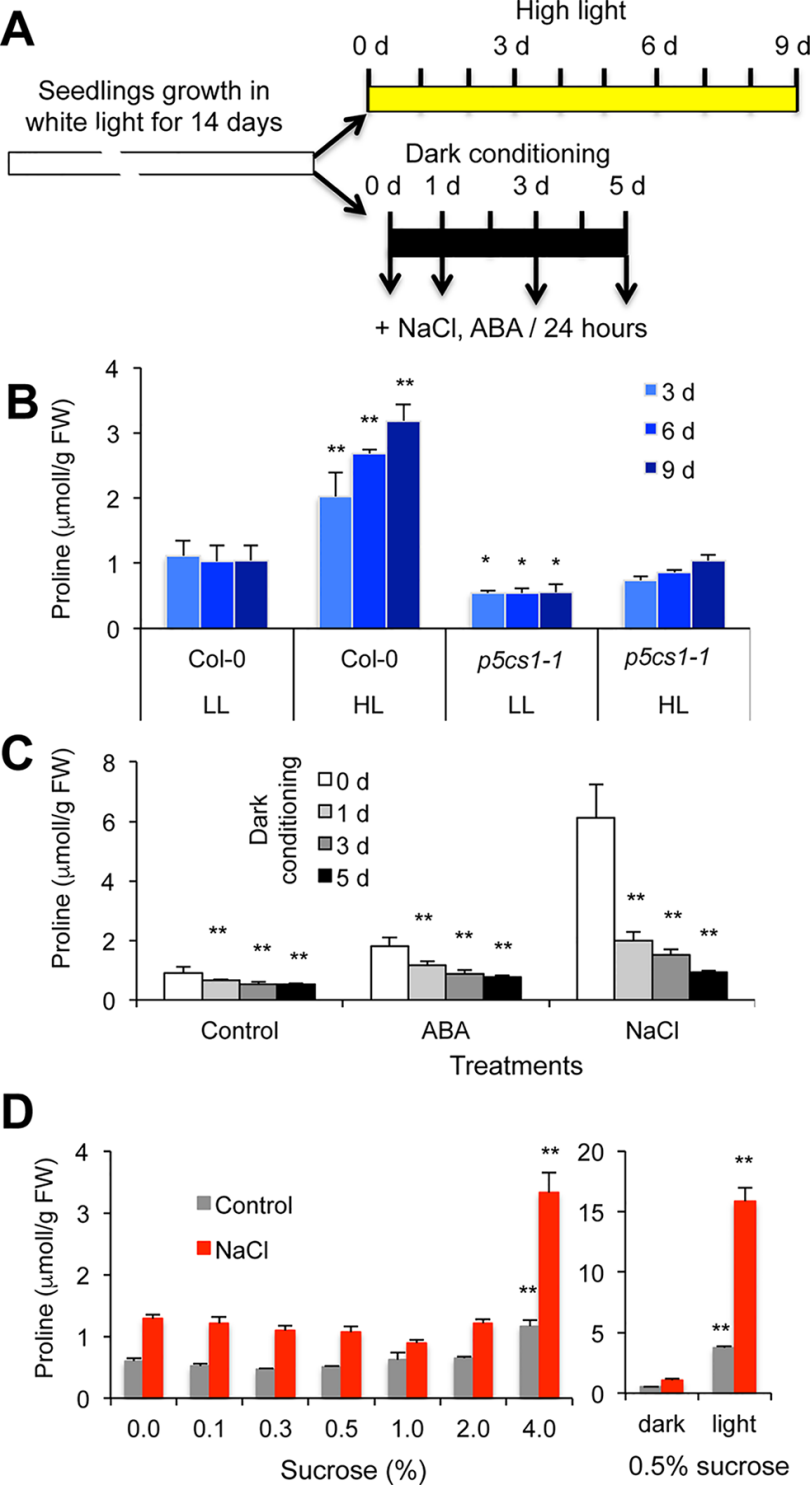
Light-dependent proline accumulation in wild-type *Arabidopsis* plants. **(A)** Experimental design. **(B)** Proline accumulation in plants after high-intensity light treatment. Plants were grown *in vitro* under standard conditions for 14 days, then transferred to high-intensity light (white light, 550 μE m^−2^ s^−1^, 8/16 h light/dark cycle) for up to 9 days. LL: low light condition, HL: high-intensity light. **(C)** Proline accumulation in dark-conditioned plants. Fourteen-day-old plantlets were transferred to dark for 1, 3, and 5 days, then subjected to salt (150 mM NaCl) or ABA (10 µM ABA) treatment in darkness for 24 h. 0 d, 1 d, 3 d, and 5 d indicates the number of days in dark. **(D)** Effect of different sugar concentrations on proline levels in dark-conditioned plants. Fourteen-day-old plants were transferred to media with different sucrose concentrations [0 to 4% (W/V)] and placed to dark for 5 days. Plants were subsequently treated with or without 150 mM NaCl and the same sugar concentrations, for 48 h. The right diagram shows proline accumulation in dark-conditioned and light-grown plants cultured on standard culture medium containing 0.5% (W/V) sucrose. Error bars indicate SD (N = 5). Significant differences compared to Col-0 plants in low light conditions **(B)**, 0 day of dark treatment **(C)** or plants cultured on standard 0.5% (W/V) sucrose **(D)** are shown: * p < 0.05, ** p < 0.01, (one-way ANOVA, Tukey test).

To characterize light-dependent proline accumulation in *Arabidopsis*, an *in vitro* experimental system was designed: Fourteen-day-old plantlets were treated with high intensity light (550 µE m^−2^ s^−1^) or deprived of light for up to 5 days, and subsequently salt or ABA-triggered proline accumulation was monitored periodically (**Figure 1A**). When plants were irradiated with strong light for several days, proline levels accumulated up to three times higher as compared to plants kept under standard light conditions (**Figure 1B**). Proline accumulation was compromised in the *p5cs1-1* mutant (Székely et al., 2008), suggesting that the *P5CS1* gene controls the rate-limiting step in proline accumulation in these conditions. In standard light conditions 1-day 10 µM ABA and 150 mM NaCl treatments lead to two or five times higher proline contents, respectively. When plants were deprived of light, proline accumulation was considerably smaller: 1 day of dark adaptation reduced proline levels from 40% to 60% of light cultured plants, whereas 5 days in darkness prevented the enhancement of proline content (**Figure 1C**). The negative effect of dark on proline accumulation may derive from the lack of adequate light or photoreceptor-derived signals reducing proline biosynthesis and/or inducing catabolism. Alternatively, if energy shortage prevents proline accumulation in dark, then externally added sugar should compensate for the absence of light. To test energy dependence, proline concentrations were measured in dark-adapted plants in the presence of various concentrations of sucrose. Proline contents in dark-adapted plants were similar in the presence of 0% and 2% (W/V) sucrose in the culture medium, whereas 4% (W/V) sucrose significantly enhanced proline accumulation. Proline levels in these conditions were, however, still far inferior to those in illuminated plants, which accumulated five to ten times more proline (**Figure 1D**). When proline content was compared in plants treated with sucrose, glucose, or mannitol, only minor differences were observed (**Figure S2**). These results suggest that sugar-dependent glycolysis cannot compensate for the lack of light signals or other photosynthesis-derived metabolites such as NADPH.

**Figure 2:**
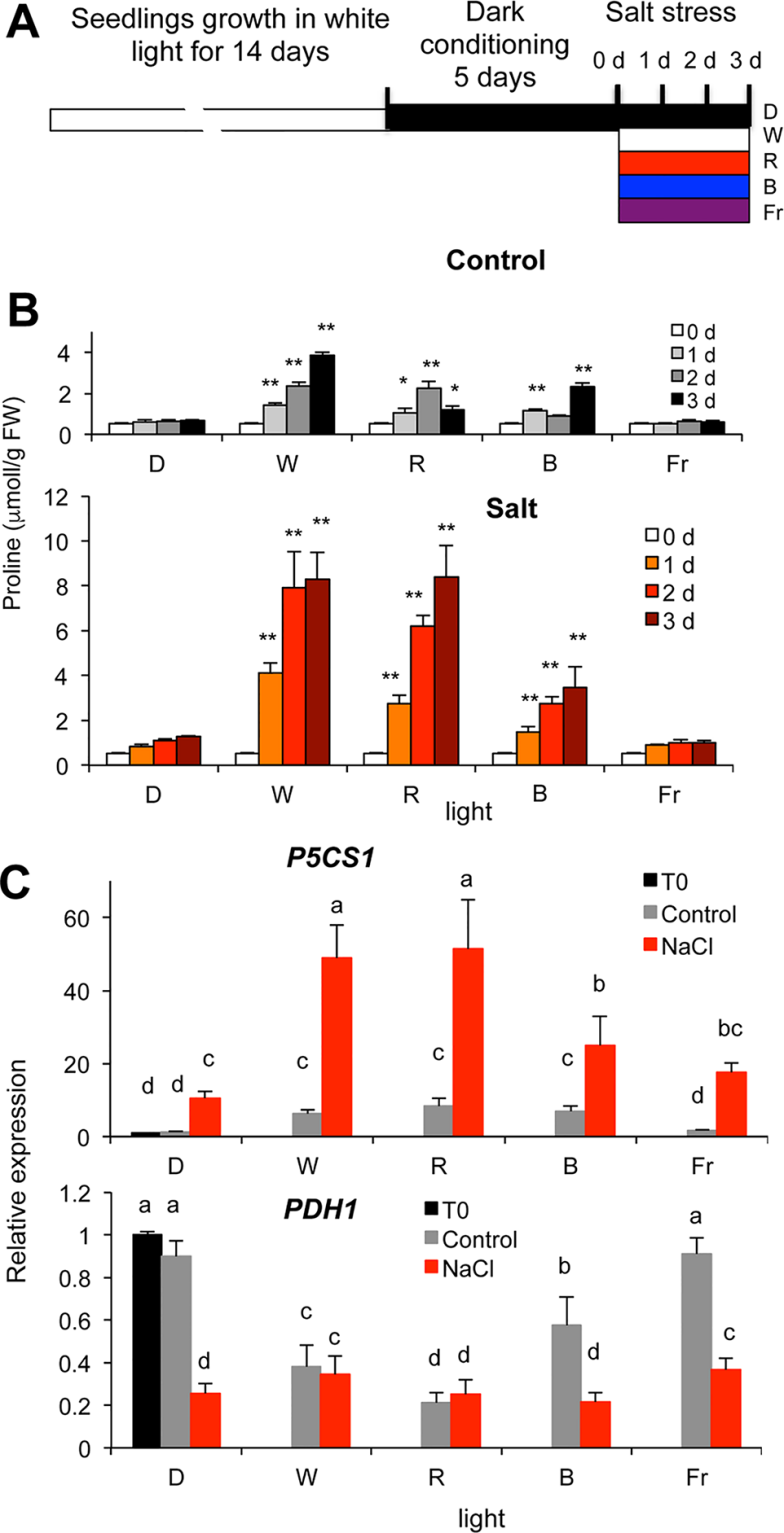
Proline accumulation in salt-treated wild-type *Arabidopsis* plants under different light regimes. **(A)** Experimental design. **(B)** Proline accumulation in dark-conditioned plants, which were subsequently kept in dark (D), illuminated by white (W), monochromatic blue (B), red (R), or far red (Fr) light, with or without salt treatment (150 mM NaCl) for up to 3 days. Significant differences to dark samples are shown (N = 6): * p < 0.05, ** p < 0.01 (one-way ANOVA, Tukey test). **(C)** Expression of the *P5CS1* and *PDH1* genes in dark-conditioned plants treated with or without 150 mM NaCl for 1 day, under different light conditions (see above). Relative transcript levels are shown, normalized to *ACT2* and *UBQ1* as well as to dark-conditioned plants, where 1 corresponds to transcript level at 0 day (T0). Error bars indicate SD (N = 3). Significant differences between means are shown by different letters (p < 0.05, one-way ANOVA, Tukey test).

Light generates specific signals, which can trigger biosynthetic and/or repress catabolic pathways. Light signals are perceived by specific photoreceptors, each of them possessing well-defined sensitivity to a particular spectrum of light (Franklin and Quail, 2010; Chaves et al., 2011). To test the effect of light quality on proline metabolism, dark-conditioned plants were transferred to white or monochromatic red, far-red or blue lights with or without simultaneous salt stress (**Figure 2A**). The light intensities for each light qualities (white light: fluorescent cool white, 4200 K, 60 µE m^−2^ s^−1^, monochromatic blue: 470 nm, 15 µE m^−2^ s^−1^, red: 660 nm, 15 µE m^−2^ s^−1^, or far-red: 730 nm, 5 µE m^−2^ s^−1^ light), were sufficient to saturate photoreceptors and light signaling cascades but not the photosynthetic electron transport (Wolf et al., 2011; Adam et al., 2013). In the absence of salt stress, proline levels increased under white, red and blue light, but remained unchanged in darkness or under far-red light (**Figure 2B**). Proline concentrations increased more than 10-fold in salt-treated plants under white or red light, whereas under blue light 5-fold enhancement was measured. Salt stress could only slightly augment free proline content in plants kept in dark or illuminated by monochromatic far-red light (**Figure 2B**). The effect of white and monochromatic light on proline accumulation was similar in Columbia 0 (Col-0) and Wassilewskija (WS) ecotypes (**Figure S3**). These results suggest that to promote proline accumulation, red is the most efficient component of the light spectrum followed by blue, whereas far-red light is insufficient.

To investigate the molecular background of light-dependent proline accumulation, expression patterns of the key metabolic genes *P5CS1* and *PDH1* were monitored under different light regimes with or without salt treatment. Transcript levels of *P5CS1* were significantly higher in plants illuminated with white, red, and blue light than in plants kept in dark or illuminated by far-red light. Salt treatment enhanced *P5CS1* expression in all light conditions, and transcript levels were highest under white and red lights followed by blue, but were moderately enhanced in far-red light or in darkness (**Figure 2C**, **Figure S4**). *PDH1* expression was downregulated by white, red, and blue light and not affected significantly under far-red light. Salt treatment repressed *PDH1* expression even more in most light conditions, including in darkness (**Figure 2C**, **Figure S4**). These results suggest that besides white light, red, and blue monochromatic lights are efficient in promoting *P5CS1* and suppressing *PDH1* expression, which ultimately leads to high levels of proline accumulation when plants are exposed to salt stress.

In order to investigate whether photosynthetic electron transport is required for proline accumulation, leaves were treated with 3-(3′,4′-dichlorophenyl)-1,1-dimethylurea (DCMU) in combination with salt (**Figure S5**). DCMU binds irreversibly to the acceptor side of photosystem II thereby inhibiting linear electron transport, which results in altered fast chlorophyll *a* fluorescence (OJIP) kinetics. Upon full inhibition, the J (F_2ms_) step equals the maximum fluorescence (F_M_ or P) intensity (Tóth et al., 2007), as seen also in **Figure S5C**. The DCMU treatment alone had no effect on proline levels, whereas NaCl-induced proline accumulation was significantly reduced (**Figure S5A**). In the presence of DCMU *P5CS1* induction was slightly reduced in salt-treated plants, whereas *PDH1* was upregulated in both salt-treated and control plants (**Figure S5B**).

### HY5 Binds to Promoter Elements of the *P5CS1* and *PDH1* Genes

**Figure 3:**
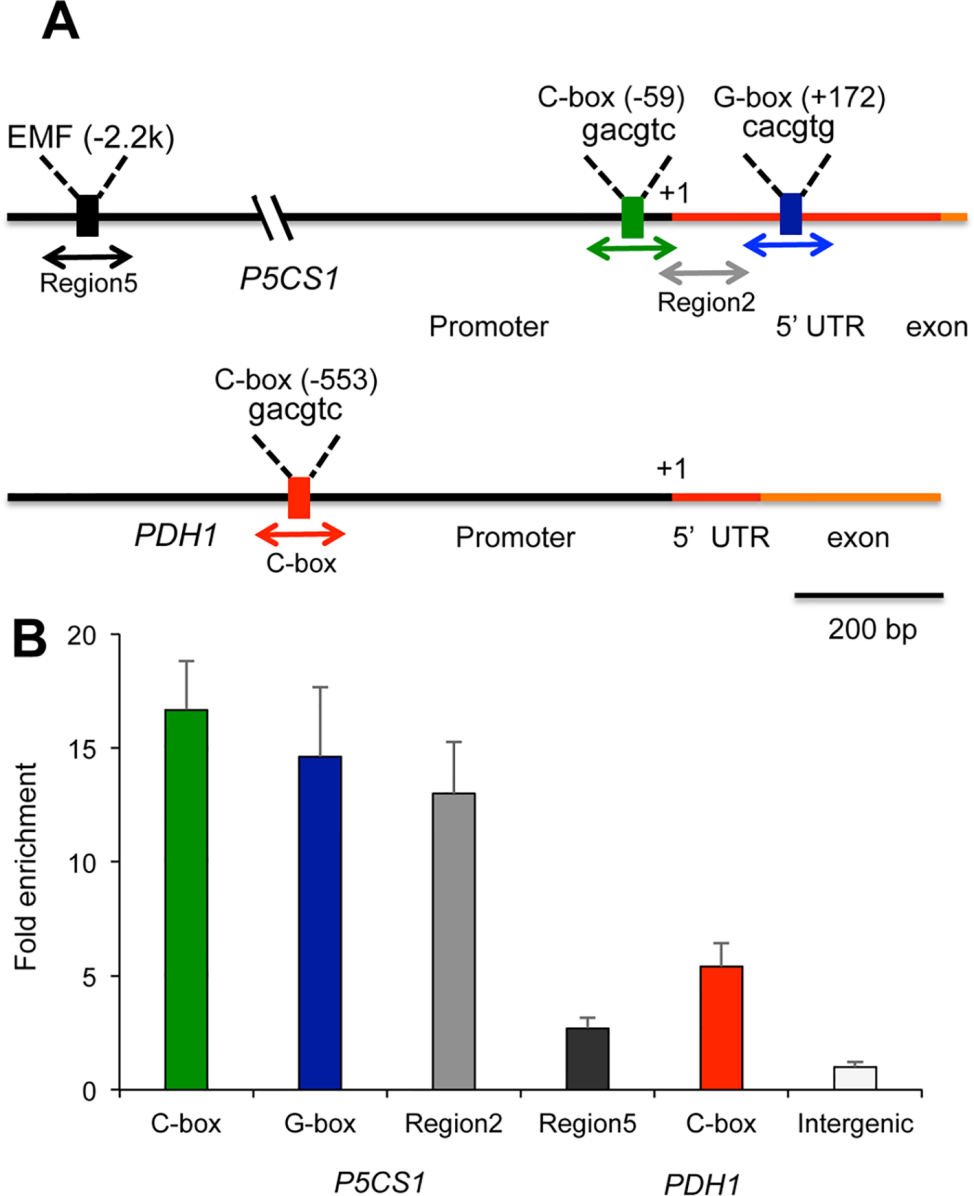
Binding of HY5 on promoter regions of the *P5CS1* and *PDH1* genes. **(A)** Schematic structure of the *P5CS1* and *PDH1* promoters indicating the positions of conserved G and C box elements and Region 2 and Region 5, according to (Feng et al., 2016). Color code: black line: promoter, red line: 5′UTR region, yellow line: exon. Boxes indicate the positions of predicted basic leucine zipper (bZIP) binding sites: black: “Essential for Memory Fragment” (EMF) (Feng et al., 2016), green: C-box, blue: G-box (*P5CS1* promoter), red box on *PDH1* promoter indicate C-box (AthaMap, http://www.athamap.de). Positions indicate distance from transcription start site (+1). Double arrows indicate the regions amplified by quantitative PCR (qPCR) after chromatin immunoprecipitation. **(B)** Result of chromatin immunoprecipitation followed by quantitative PCR (ChIP-qPCR) tests on two *P5CS1* and one *PDH1* promoter region. An intergenic region with no C or G-box sequences was used as reference (= 1). Error bars on diagrams indicate SD (N = 3).

The bZIP-type transcription factor HY5 is a key positive regulator of light-dependent gene expression that controls transcription of thousands of light-induced genes (Cluis et al., 2004; Toledo-Ortiz et al., 2014). A recent ChIP-seq analysis of HY5 binding sites revealed that this transcription factor recognizes conserved *cis*-acting elements in more than three thousand genes, both under red and blue light (Hajdu et al., 2018). Genome-wide mapping of HY5 binding sites revealed that this TF can recognize the promoter regions of the *P5CS1* and *PDH1* genes (**Figures S6**, **S7**). While peak of the reads were mapped close to the transcription initiation site of *P5CS1*, maximum reads were localized to 0.5 kb upstream of the *PDH1* transcription initiation site. Another recent study revealed binding of HY5 to the 5′ UTR and a distal upstream region of *P5CS1* (Feng et al., 2016). Promoter and 5′ UTR regions of *P5CS1* contain a number of predicted regulatory sequence motifs, including a G-box (CACGTG) at +172 bp in the 5′ UTR and a C-box (GACGTC) in the promoter, at −59 bp distance from the transcription start site, which can serve as binding sites of HY5 (**Figure 3A**) (Fichman et al., 2015). The *PDH1* promoter contains one conserved C-box motif in the promoter, at −553 bp distance from the transcription start site (**Figure 3A**).

To verify HY5 binding in the identified regulatory regions, ChIP followed by quantitative PCR (ChIP-qPCR) assays were performed on *P5CS1* and *PDH1* promoter fragments containing the predicted G-box and C-box sequence motifs. When compared to intergenic regions, specific enrichment in qPCR-amplified C-box- and G-box-containing *P5CS1* and *PDH1* promoter fragments was detected in the HY5-YFP-immunoprecipitated DNA samples (**Figure 3B**). Enrichment of HY5 binding to *P5CS1* G-box and C-box regions was around 14 to 16 times higher while it was 5 times higher on C-box region of the *PDH1* promoter than on a control intergenic region (**Figure 3B**). ChIP-qPCR experiments therefore confirmed that HY5 interacts *in vivo* with the selected 5′ UTR and promoter regions of both the *P5CS1* and the *PDH1* genes. To compare our results with previously reported HY5 assays, ChIP assay was performed with primers used to amplify Region 2 and Region 5 of *P5CS1*, as defined by Feng et al. (2016). Region 2 is a 140 bp fragment, in the 5′ UTR (from −5 to +135 bp), flanked by C-box and G-box sequences. Region 5 is a 147 bp fragment in the upstream region (from −2129 to −2276 bp), which corresponds the previously described “Essential for Memory Fragment” (EMF) (Feng et al., 2016). In our experimental conditions enrichment of Region 2 in ChIP assay was similar to fragments containing G-box and C-box elements, whereas enrichment was a magnitude lower when HY5 binding was tested for Region 5, corresponding to EMF (**Figure 3B**).

**Figure 4:**
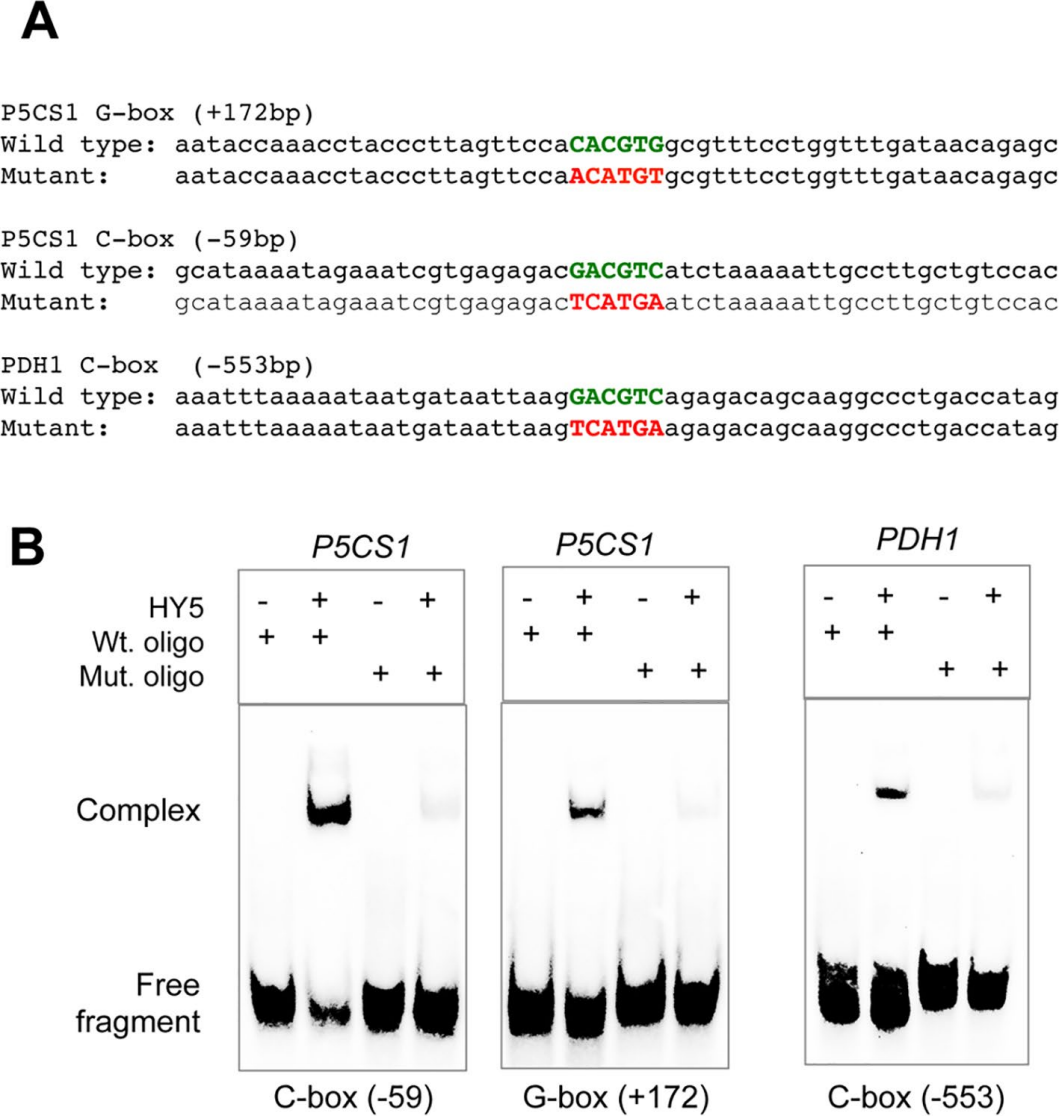
Confirmation of TF binding sequence motifs with electrophoretic mobility assay (EMSA). **(A)** Sequences of the oligonucleotides containing wild-type and mutated G-box and C-box sequences of the *P5CS1* and *PDH1* promoters. **(B)** EMSA assays with wild-type (Wt. oligo) and mutant (Mut. oligo) double stranded oligonucleotides and purified HY5 protein. Note complex formation of HY5 protein with wild-type oligonucleotides, which is almost invisible with mutant ones with altered ACGT core sequences.

To verify that the conserved C-box and G-box motifs are indeed the targets of HY5, electrophoretic mobility shift assays (EMSA) were performed using 56 bp dsDNA fragments containing the native regulatory sequences or their mutated forms in which the conserved CACGTG or GACGTC sequence motifs were altered, eliminating the core ACGT sequence (**Figure 4A**). Complex formation of HY5 with ds oligonucleotides corresponding to wild-type *P5CS1* and *PDH1* promoter fragments was observed in the EMSA assays. Complexes between HY5 and oligoes carrying the mutated G-box or C-box sequences were, however, not formed or were detected at much lower level (**Figure 4B**). These experiments confirmed that the C-box and G-box sequences are indeed responsible for HY5 binding to the *P5CS1* promoter (−59 bp) or the 5′ UTR (+172 bp) regions as well as binding of the *PDH1* promoter (−553 bp) region.

### HY5 Regulates Proline Accumulation and Expression of the *P5CS1* and *PDH1* Genes

**Figure 5:**
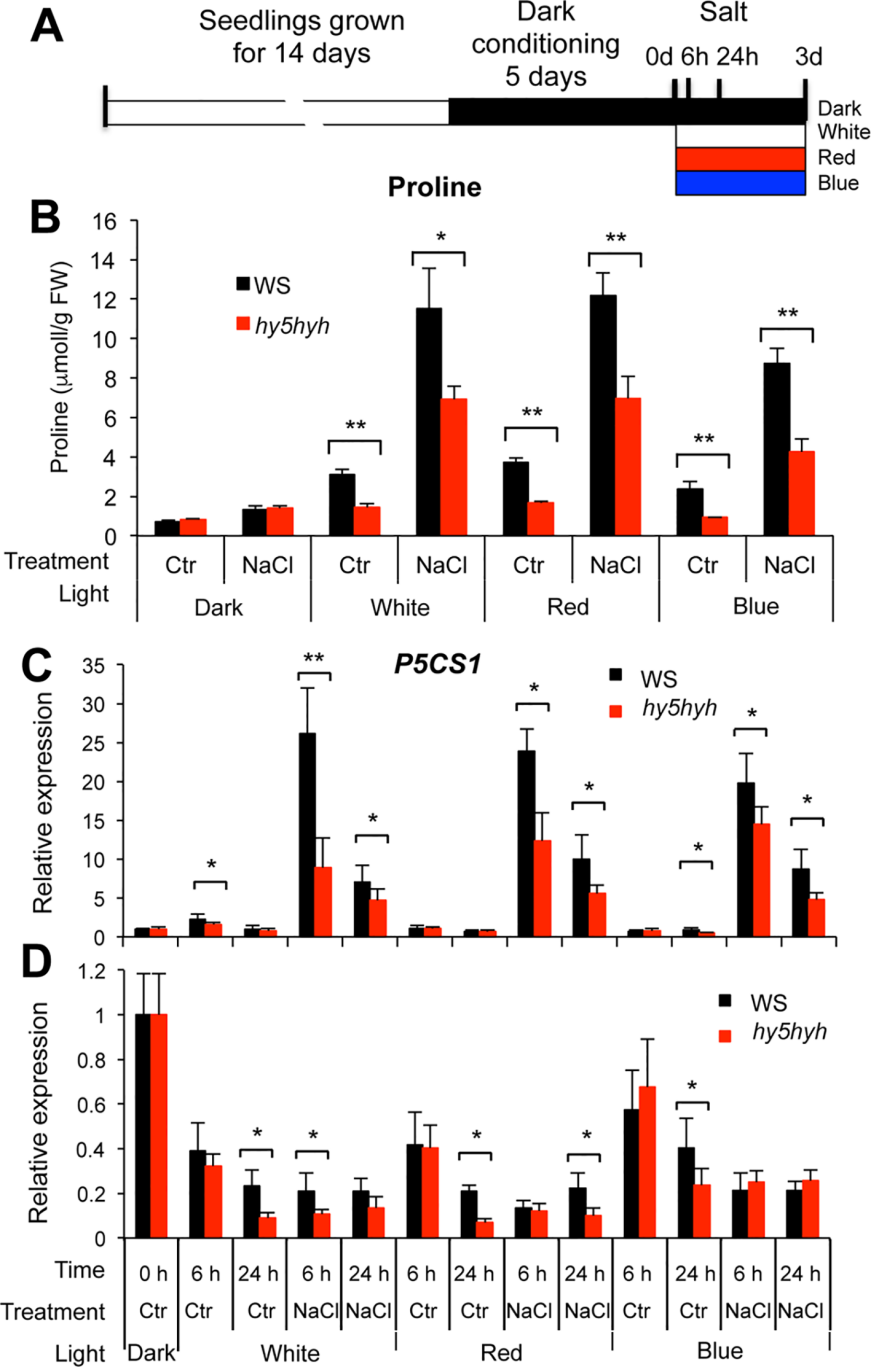
Proline accumulation and expression of the *P5CS1* and *PDH1* genes in salt-treated wild-type and *hy5hyh* double mutant plants. **(A)** Experimental design: 14-day-old *in vitro*-grown plants were conditioned by dark treatment for 5 days and subsequently treated with or without 150 mM NaCl and illuminated with white or monochromatic red or blue light. **(B)** Proline accumulation in Wassilewskija (WS) wild-type and *hy5hyh* mutant plants after 3 days of salt treatment. Error bars indicate SD (N = 5). **(C**, **D)** Transcript levels of the *P5CS1***(C)** and *PDH1***(D)** genes after 6 and 24 h of salt treatment. Relative transcript levels are shown, which were normalized to *ACT2* and *UBQ1* reference genes as well as to dark-conditioned plants. Abbreviations: Ctr: control, NaCl: salt treatment. Error bars indicate SD (N = 3). Significant differences between wild type and mutant values are: * p < 0.05, ** p < 0.01 (two-way ANOVA, Tukey test, fixed parameters were genotypes and treatments).

To study the function of HY5 in proline metabolism, free proline contents and transcript levels of *P5CS1* and *PDH1* genes were compared in wild-type (WS) and *hy5hyh* mutant plants carrying knockout mutations for both *HY5* and the closely related *HYH* genes (Hajdu et al., 2018). Wild-type and *hy5hyh* double mutant plants were conditioned to dark as described above, and were subsequently treated by salt under white and monochromatic red or blue light (**Figure 5A**). Compared to plants kept in darkness, proline levels were enhanced by illumination with both white and red or blue monochromatic lights. When compared to wild type, proline levels were not affected or were slightly lower in illuminated *hy5hyh* mutants without salt treatment (**Figures 5B**, **S8**). Salt stress enhanced proline contents three to six times in illuminated plants, while proline accumulation was around 50% lower in the *hy5hyh* mutant when compared to wild-type plants in the same conditions (**Figures 5B**, **S8**). When plants were kept in darkness, proline levels were only slightly increased by salt treatment, and enhancement was similar in both genotypes.

*P5CS1* transcript levels were low in both wild-type and *hy5hyh* mutants without salt treatment with minor induction by illumination. *P5CS1* expression was clearly induced by 6 h of salt treatment in illuminated plants, reaching approximately 50% lower transcripts in the *hy5hyh* mutant than in wild-type plants (**Figures 5C**, **S9B**). In all light conditions transcript levels were higher after 6 h of stress than after 24 h (**Figure 5C**). *PDH1* expression was reduced by illumination and by salt stress in all light conditions. Genotype-dependent differences in *PDH1* transcript levels were however less pronounced and downregulation was more variable (**Figures 5D**, **S9C**). The *P5CS1* transcription pattern positively correlated with changes in proline levels, indicating that biosynthesis is essential in defining proline accumulation in these conditions, while *PDH1*-controlled catabolism can fine-tune proline levels. These data indicate that HY5 (and possibly HYH) is a positive regulator of proline accumulation by contributing to the expression of the *P5CS1* gene with a minor role in the control of *PDH1* expression.

## Discussion

**Figure 6:**
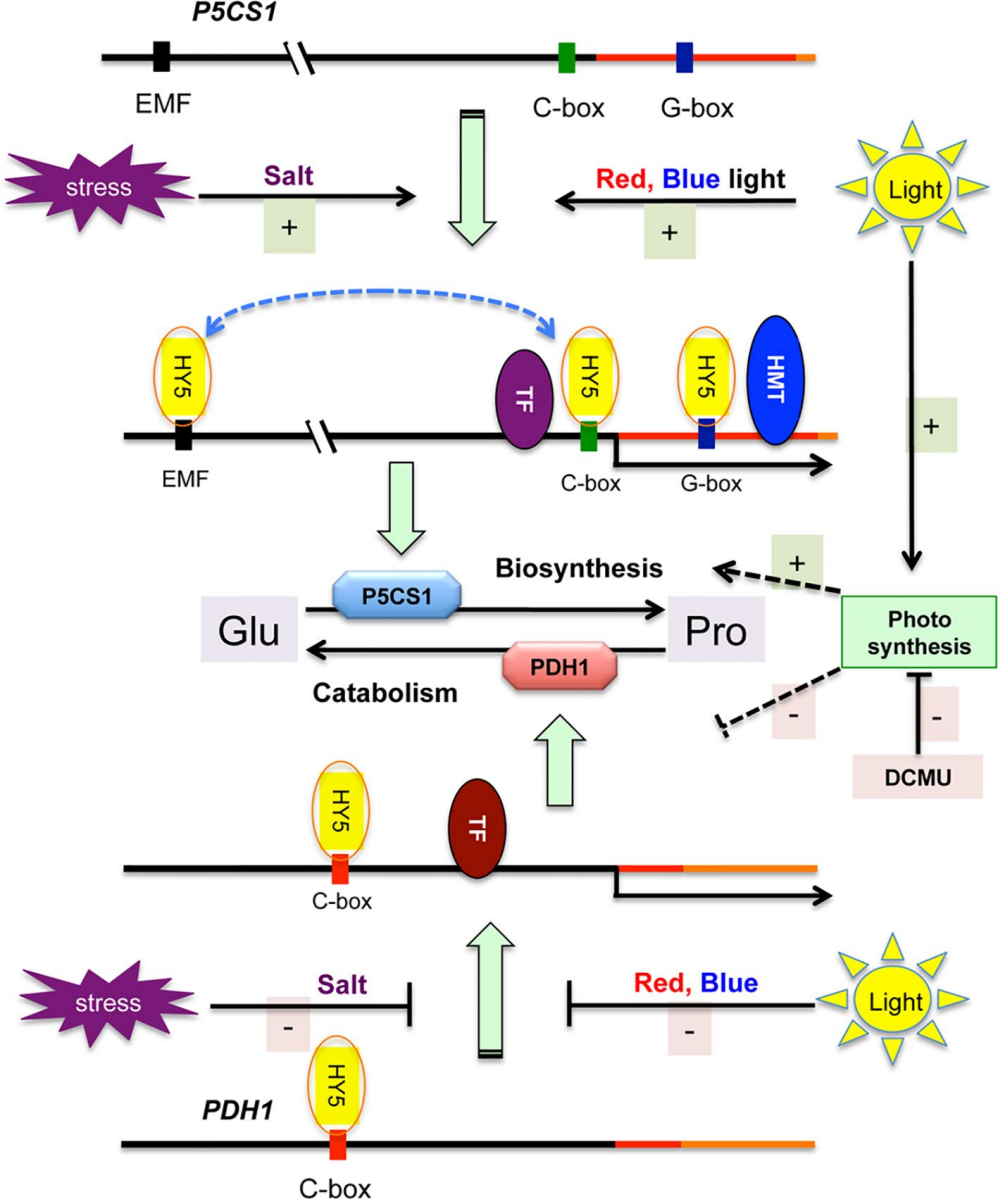
Model of stress and light regulation of proline metabolism in *Arabidopsis*. Salt stress and light induces *P5CS1* and inhibits *PDH1* expression, promoting proline biosynthesis and reducing catabolism. Schematic maps of *P5CS1* (upper line) and *PDH1* (lower line) promoter and 5′ UTR regions are shown. Only HY5 binding sites are indicated in the schematic maps. HMT corresponds to histone merthyltransferase and TF indicates other transcription factors which can regulate transcription of *P5CS1* (Feng et al., 2016; Aleksza et al., 2017; Fu et al., 2018) or *PDH1* (Satoh et al., 2004; Weltmeier et al., 2006; Dietrich et al., 2011). Light-controlled HY5 binds to the promoters of both genes and contributes to *P5CS1* activation, but has only a minor effect on *PDH1* expression. Stress conditions as well as red and blue light activate *P5CS1* transcription (upper scheme), and inhibits *PDH1* activation (lower segment of the scheme). Photosynthesis can promote proline accumulation (probably enhancing biosynthesis and reducing catabolism) *via* an unknown mechanism, which is inhibited by 3-(3′,4′-dichlorophenyl)-1,1-dimethylurea (DCMU). Solid lines indicate confirmed, dashed lines show unknown/predicted interaction or regulation.

In this study we investigated the importance of light in salt-dependent proline accumulation, focusing on HY5-mediated light signals. A model summarizes our results integrating it with previous studies (**Figure 6**). Light was previously shown to promote proline accumulation and inversely influence *P5CS1* and *PDH1* expression in *Arabidopsis* plants (**Figure S1**, Hayashi et al., 2000; Abraham et al., 2003). Here we showed that high light enhances, while extended darkness reduces proline levels, and absence of light cannot be compensated by externally supplied sugar as energy source (**Figures 1**, **S2**). Proline metabolism in *Arabidopsis* was found to be controlled by red and blue lights but is less influenced by far red light (**Figures 2**, **S3**). We showed that light-dependent proline accumulation is regulated by HY5, a key bZIP-type transcription factor in light signaling which is known to be a positive regulator of photomorphogenesis (**Figures 5**, **S8**, **S9**, Holm et al., 2002; Toledo-Ortiz et al., 2014). Genome-wide ChIP-chip or ChIP-seq experiments revealed that HY5 directly controls around 10% of the *Arabidopsis* genes through binding to their promoters (Lee et al., 2007; Zhang et al., 2011; Hajdu et al., 2018). Datamining of the ChIP-seq supplementary datasets revealed that HY5 recognizes the 5′ regions of the *P5CS1* and *PDH1* genes, suggesting that these genes are direct targets of this bZIP factor (Hajdu et al., 2018) (**Figures S6**, **S7**). HY5 was found to bind directly to the promoter or 5′ UTR regions of the key metabolic genes *P5CS1* and *PDH1*, which modulate rate-limiting steps in proline biosynthesis and degradation. The 5′ regulatory region of *P5CS1* contains various *cis*-regulatory elements including a well-defined G-box in the 5′UTR region and a C-box motif in the promoter, which are conserved in *P5CS1* promoters of closely-related Brassicaceae species (Fichman et al., 2015). Sequence analysis revealed one C-box motif in the *PDH1* promoter. ChIP-qPCR experiments demonstrated *in vivo* binding of HY5 to at least three promoter regions of *P5CS1*, and one region of *PDH1*, which contained G-box or C-box sequence elements (**Figure 3**). EMSA experiments demonstrated that HY5 can directly and specifically bind to these sequence motifs *in vitro* (**Figure 4**). Promoter binding therefore strongly suggest that HY5 is directly involved in the control of *P5CS1* and *PDH1* transcription. G-box and C-box sequence motifs have an ACGT core, which is essential for binding of bZIP transcription factors, whereas nucleotides flanking the core sequence define the specificity of sequence recognition (Williams et al., 1992; Izawa et al., 1993). Mutations eliminating the ACGT core in the *P5CS1* and *PDH1* G-box and C-box motifs weakened or abolished HY5 binding to these DNA fragments, confirming that these promoter elements are indeed critical for the complex formation with this transcription factor (**Figure 4**). ACGT-containing sequence motifs are present in ABA Response Elements (ABRE), binding sites of bZIP-type AREB/ABF type transcription factors, which are key regulators of ABA-induced gene activation (Hobo et al., 1999; Fujita et al., 2005; Yoshida et al., 2010). Polymorphism in ABRE or adjacent CE motifs were recently shown to influence *P5CS1* expression and proline accumulation in barley, although TF binding to these motifs was not reported (Muzammil et al., 2018).

Two other HY5 binding regions were previously identified in the *Arabidopsis P5CS1* promoter, which were implicated in maintaining stress memory (Feng et al., 2016). Enhanced H3K4me3 levels near the *P5CS1* transcription start site were associated with light exposure and shown to correlate with transcript levels in repeated stresses. The distal EMF (Region 5 in **Figure 3**) is located 2.2 kb upstream of the transcription start site, and contains a C/A box, which can bind HY5 (Feng et al., 2016). Region 2 is located in 5′ UTR, which however has no recognizable sequence element for HY5 binding, but is flanked by the G-box and C-box sequence motifs, reported in this study. In our ChIP-qPCR assay similar degrees of enrichments were detected for G-box, C-box motifs and Region 2 of Feng et al. (2016) (**Figure 3**). ChIP technology has 300 to 400 bp resolution which can cover Region 2 and the flanking G-box and C-box motifs. ChIP-qPCR with primers in Region 2 could therefore detect chromatin fragments which were immunoprecipitated by the flanking G-box or C-box elements. Similar degrees of ChIP-qPCR enrichment were reported earlier for both Region 2 and Region 5 (Feng et al., 2016). In our ChIP assay binding efficiency of HY5 to Region 5 was however a magnitude lower than binding to C- or G-boxes (**Figure 3**). We used functional YFP-tagged HY5 and GFP-trap agarose beads for ChIP, whereas Feng et al. (2016) employed anti-HY5, which may explain the differences.

One of the key steps in the regulation of light-dependent gene expression is the photoreceptor-induced accumulation of the HY5 protein. Since this process is affected by virtually all photoreceptors, *hy5* mutants show photomorphogenic phenotypes, such as elongated hypocotyls not only in blue or red, but in far-red light as well (Abbas et al., 2014). Our data demonstrated that stress-induced proline accumulation did not occur in far-red light. One possibility is that far-red-derived signals are insufficient for *P5CS1* induction and proline accumulation during salt stress. PHYA is the sole light receptor in *Arabidopsis* that can be activated by far-red light (Casal et al., 2014). Although PHYA signaling promotes the accumulation of HY5, apparently alone it cannot activate *P5CS1* transcription. Alternatively, low photosynthetic activity under far red light might prevent proline biosynthesis and promote catabolism *via* metabolic regulation (eg. due to low NAPDH pools) (Thapper et al., 2009; Pavlou et al., 2018).

Heterodimerization of bZIP transcription factors allows combinatorial control of target gene expression (Ehlert et al., 2006; Yoshida et al., 2010; Dietrich et al., 2011). HY5 and the related HYH factors were shown to form homo and heterodimers and promote light-induced expression of target genes (Holm et al., 2002). Formation of G-box-binding heteromers of HY5 with other bZIP factors was reported, suggesting that this transcription factor may cooperatively regulate transcription of ABA-induced target genes with other bZIP factors such as ABFs (Yoshida et al., 2010; Singh et al., 2012). Whether HY5 interacts with ABFs or other TFs on *P5CS1* and/or *PDH1* promoters remains to be elucidated.

The functionality of promoter binding by HY5 was tested by comparing transcript levels of the *P5CS1* and *PDH1* genes and proline accumulation in *hy5hyh* double mutant with those in wild-type plants under different light regimes (**Figures 5**, **S8**, **S9**). HY5 and HYH transcription factors are partially redundant, therefore the double knockout *hy5hyh* mutant was used in these studies. *P5CS1* transcript levels were lower in the salt-treated *hy5hyh* double mutant, suggesting that HY5 and perhaps the closely related HYH indeed contribute to high-level *P5CS1* induction in salt-stressed plants. Lower proline levels in salt-treated *hy5hyh* plants correlated with reduced transcript levels of *P5CS1*. Expression of *PDH1* was less influenced in the *hy5hyh* mutant, although minor differences could be detected during illumination with monochromatic light. These results confirm the positive role of HY5 in proline accumulation, which mediates light signals and modulates transcriptional activities of key metabolic genes. Complex formation of HY5 with the distal EMF region (Feng et al., 2016) and 5′ UTR sequences of *P5CS1* promoter was required for the retention of H3K4me3 levels and the maintenance of stress memory (Feng et al., 2016). HY5 seems to functions as a regulatory hub, which transmits light signals and connects them to stress and/or ABA signals and histone methylation and regulates the transcription of key metabolic genes by directly binding to conserved *cis* regulatory elements of their promoters (**Figure 6**).

In addition to photoreceptor-mediated signaling, light may modulate proline biosynthesis in other ways. Light can provide energy and reducing agents such as NADPH through photosynthesis, and light can modulate gene expression by specific signals, such as the redox state of the plastoquinone pool. The decline of proline levels in darkness could not be compensated by externally added sugars, suggesting that energy limitation is not a principal reason of light dependency (**Figures 1**, **S2**). Inhibition of photosynthetic electron transport with DCMU, however, reduced salt-dependent proline accumulation, *P5CS1* activation, and considerably promoted *PDH1* expression, demonstrating that photosynthesis itself can influence proline metabolism (**Figure S5**). Previously P5CS1-GFP was localized in chloroplasts in salt-treated cells, supporting the assumption that proline biosynthesis can be associated with photosynthesis in stress conditions (Székely et al., 2008; Szabados and Savoure, 2010; Sharma et al., 2011). Glutamate-derived proline biosynthesis is a reductive metabolic pathway, which could be stimulated by photosynthetic NADPH in osmotically stressed *Lotus corniculatus* leaves (Diaz et al., 2005). Alternatively, chloroplast to nucleus retrograde signaling could be implicated in light control of *P5CS1* and *PDH1* genes (Gollan et al., 2015; Kleine and Leister, 2016). Deciphering the exact mechanism how light and photosynthesis regulates proline metabolism, however needs further investigation.

## Materials And Methods

### Plant Material and Growth Conditions

*Arabidopsis thaliana* plants were either Col-0 or WS ecotype. The *hy5hyh* double mutant (Holm et al., 2002) has WS background. Basic conditions of plant growth were described before (Aleksza et al., 2017). Briefly: seeds were surface sterilized and germinated on 1/2MS culture medium containing 0.5% (W/V) sucrose. Plants were grown *in vitro* in growth chambers under 120 µE m^−2^ s^−1^ illumination (white light) using a 8 h light/16 h dark cycle, and 22°C/18°C temperature cycle for 14 days.

For high-intensity light treatment 14-day-old plants were illuminated with white light with 550 µE m^−2^ s^−1^ light intensity in growth chambers. For dark conditioning, 14-day-old plantlets were transferred to dark, and incubated in the absence of light for up to 5 days in the same conditions (medium, temperature). For subsequent light induction, plants were transferred to either white light (fluorescent cool white, 4200 K, 60 µE m^−2^ s^−1^), or monochromatic blue (470 nm, 15 µE m^−2^ s^−1^), red (660 nm, 15 µE m^−2^ s^−1^), or far-red (730 nm, 5 µE m^−2^ s^−1^) light, or kept in dark for up to three further days. The primary criteria for setting the fluence rate of light was to reach saturation of signaling cascades triggered by phytochrome and cryptochrome photoreceptors. The most studied light responses are saturated at the light intensities described above (Wolf et al., 2011; Adam et al., 2013). Dark-conditioned plants (including those kept in constant darkness) were transferred and handled under green light.

To induce proline accumulation, plants were cultured on the surface of thin-layer liquid culture medium (10 ml medium/13 cm diameter Petri dish), using nylon mesh to prevent submergence. For stress, liquid media were supplemented with 150 mM NaCl or treated with 10 µM ABA. Proline levels were determined in plants for up to 3 days as described.

### Proline Measurements

Proline content was determined by the ninhydrin-based coloritmetric method as described (Abraham et al., 2010). Alternatively, a microtiter-scale colorimetric reaction was used, which was based on a recent paper (Lee et al., 2018) with some modifications. Plant material (approximately 50 mg fresh weight/sample) was ground and 20 µl of 1% (W/V) sulfosalicylic acid was added per mg FW tissue. After centrifugation at top speed (15.000 rpm) for 5 min at 4°C in a microcentrifuge, the supernatant was removed and mixed with acidic ninhydrin [1,25% (W/V) ninhydrin in 80% (V/V) acetic acid] in 1:2 ratio, and incubated at 95°C for 30 min. The reaction was terminated on ice, and absorbance was measured at 510 nm in a plate reader (MULTISKAN GO, Thermo Scientific) using a 1:2 mixture of sulfosalicylic acid and acidic ninhydrin as reference. The system was calibrated with standard curves with known concentrations of proline. Anthocyanine accumulation was not visible in the plants after these treatments. Experiments were repeated three times and four to six replicates were used to determine proline levels in a treatment.

### Fast Chl A Fluorescence (OJIP) Measurements

Fluorescence measurements were carried out at room temperature with a Handy-PEA instrument (Hansatech Instruments Ltd, UK). Plants were dark-adapted for 30 min and detached leaves were then placed in a modified Handy-PEA leaf clip. The leaf sample was illuminated with continuous red light (3500 µE m^−2^ s^−1^, 650 nm peak wavelength; the spectral half-width was 22 nm; the light emitted by the LEDs is cut off at 700 nm by a NIR short-pass filter). The light was provided by an array of three light-emitting diodes focused on the sample surface. The first reliably measured point of the fluorescence transient is at 20 µs, which can be taken as F_0_ (O). The length of the measurements was 1 s.

### DCMU Treatment

Fourteen-day-old *in vitro*-grown plants (Col-0 ecotype) were transferred to 150 mM NaCl and/or sprayed with 50 µM DCMU solution. OJIP fluorescence was measured 3 and 24 h after DCMU and salt treatments. Proline accumulation was measured 24, 48, and 72 h after DCMU treatment, whereas gene expression was measured after 24 h.

### Gene Expression Studies

To test transcript levels of selected genes, quantitative RT-PCR (qRT-PCR) was performed on cDNA templates obtained from total RNA samples. RNA isolation was performed with Nucleo Spin RNA isolation kit (Macherei-Nagel). Total RNA was DNase treated with TURBO DNA-free™ Kit (Invitrogen by Thermo Fisher Scientific). First-strand cDNA synthesis of 1.5 µg of total RNA was carried out with RevertAid M-MuLV Reverse Transcriptase (Fermentas), using random hexamers. Real-time PCR was carried out with the ABI 7900 Fast Real Time System (Applied Biosystems). The protocol in 45 cycles was 15 s at 95°C, followed by 1 min at 60°C. The specificity of the amplifications was verified using the ABI SDS software. Expression of the *P5CS1* (*AT2G39800*) and *PDH1* (*AT3G30775*) genes was monitored by qRT-PCR as described (Aleksza et al., 2017). Normalized transcript levels were calculated by the modified 2^−ΔΔCt^ method using averages of *actin2* (*AT2G37620*) and *UBQ1* (*AT3G52590*) Ct values as reference (Livak and Schmittgen, 2001; Vandesompele et al., 2002). In relative expression data of the figures, reference was obtained on non-treated plants at the start of the experiment (*e.g.* dark-adapted plants, just before light and stress treatments). Statistical analysis was made on 2^−ΔΔCt^ values of three replicates corresponding to cDNA templates and RNA samples isolated from three different Petri plates. Experiments were repeated at least twice. Primers used in qRT-PCR experiments are listed in **Table S1**.

The average amplification efficiencies of each primer pair used in the qRT-PCR experiments were derived from the slope of the amplification curve in the exponential phase of three different reactions from three different samples. The corresponding PCR efficiency was calculated according to the formula: E = 10 (1/slope) (Svec et al., 2015). Each primer showed high amplification efficiency from 1.99 to 2.03. Sequences of the PCR primers are available in **Table S1**.

### Chip Followed by Quantitative PCR

The ChIP protocol by Werner Aufsatz (http://www.epigenome-noe.net/researchtools/protocol.php?protid=13) was applied with the following modifications. Fourteen-day-old *hy5* mutant plants expressing HY5-YFP fusion proteins from the *HY5* promoter (Hajdu et al., 2018) were fixed in 1% (V/V) formaldehyde solution. Chromatin samples were sonicated on ice six times for 10 s using a Vibra Cell sonicator (SONICS & MATERIALS Inc., Danbury, CT, USA) at 10% power. Sonicated and diluted chromatin samples were pre-cleared by 20 µl (bed volume) of binding control agarose beads (Chromotek GmbH, Germany) for 1 h at 4°C. An aliquot of the pre-cleared chromatin solution was saved for the input sample and the rest of the material was precipitated using 12.5 µl (bed volume) of GFP-Trap agarose beads (Chromotek GmbH, Germany) for 16 h at 4°C. Precipitated chromatin was eluted from the beads, and along with the input sample, it was de-crosslinked and DNA was extracted using the Silica Bead DNA Gel Extraction Kit (Thermo Scientific). The final volume of purified DNA samples was about 45 µl. 1.5 µl of the eluate was analyzed in qPCR reactions. Primers were designed to amplify genomic regions around the putative HY5 binding sites. Standard series were prepared from 10-fold dilutions of the input DNA samples. ChIP-related qPCR primers are listed in . ChIP data were analyzed and presented according to the “percent of input” method (Haring et al., 2007). Experiments were repeated three times.

### Electrophoretic Mobility Assay

The pET28a vector carrying the full-length HY5 cDNA fragment (Hajdu et al., 2018) was introduced into *Escherichia coli* BL21 DE3 Rosetta cells (New England 513 Biolabs). Proteins were purified on His-Select Nickel affinity gel (SIGMA). 2 µg of purified protein was incubated for 30 min with 2 pmol biotin-labeled DNA (respective P5CS1 and PDH1 oligonucleotide sequences are available in ). DNA fragments and complexes were separated in 4% (W/V) native polyacrylamide gel, then blotted to HyBond-N^+^ nucleic acid transfer membrane (Amersham). DNA fragments were crosslinked to the membrane with UV light (UV Stratalinker, Stratagene). DNA fragments were detected by an immune reaction with Streptavidin-conjugated horseradish-peroxidase (Thermo Scientific) using the LightShift Chemiluminescent EMSA Kit (Thermo Scientific). Signals were developed with a chemiluminescent substrate (Supersignal West-Thermo Scientific) and detected in Fusion FX western blot and *gel documentation* imaging device (Vilber). Experiments were repeated twice.

### Informatics, Statistical Analysis

Promoter sequence analysis was performed with AthaMap tool (http://www.athamap.de). Oligonucleotides were designed and analyzed by IDT OligoAnalyzer (https://eu.idtdna.com/calc/analyzer). Oligonucleotides used in this study are listed in Table S1.

Statistical analyses (one-way and two-way ANOVA, means comparisons by Tukey tests) were performed using the OriginPro 2018 software version 9.5 (OriginLab Corporation, Northampton, MA, USA). In case of one-way ANOVA the differences between means were determined Tukey test or by Duncan’s multiple range test and labeled in all diagrams by different letters. When two-way ANOVA was used, the means comparison were made with Tukey test. Data were processed and in some experiments Diagrams were prepared with MS Excel 14.7.7, and figures were assembled with MS Powerpoint 14.7.7 and Adobe Photoshop CS5.1.

## Author Contributions

HK, DA, AIB, AH, AK, LZ, and ST performed the experiments. LK-B evaluated the results and corrected the manuscript. LS directed the research and wrote the manuscript.

## Funding

This research was supported by NKFI Grants K128728, NN118089, and GINOP Project nos. 2.3.2-15-2016-00001 and 2.3.2-15-2016-00023. HK was supported by the Young Scientist Fellowship of the Hungarian Academy of Sciences.

## Conflict of Interest

The authors declare that the research was conducted in the absence of any commercial or financial relationships that could be construed as a potential conflict of interest.
