# Supplementary material for: Light Control of Salt-Induced Proline Accumulation Is Mediated by ELONGATED HYPOCOTYL 5 in Arabidopsis
Source: Front Plant Sci. 2019 Dec 10;10:1584. doi: 10.3389/fpls.2019.01584 (PMC6914869; doi:10.3389/fpls.2019.01584)
Supplement: Supplementary file 1 [file Presentation_1.pdf]

Figure S1. Daily fluctuation of *P5CS1*, *P5CR* and *PDH1* transcripts in Arabidopsis in mild drought conditions.

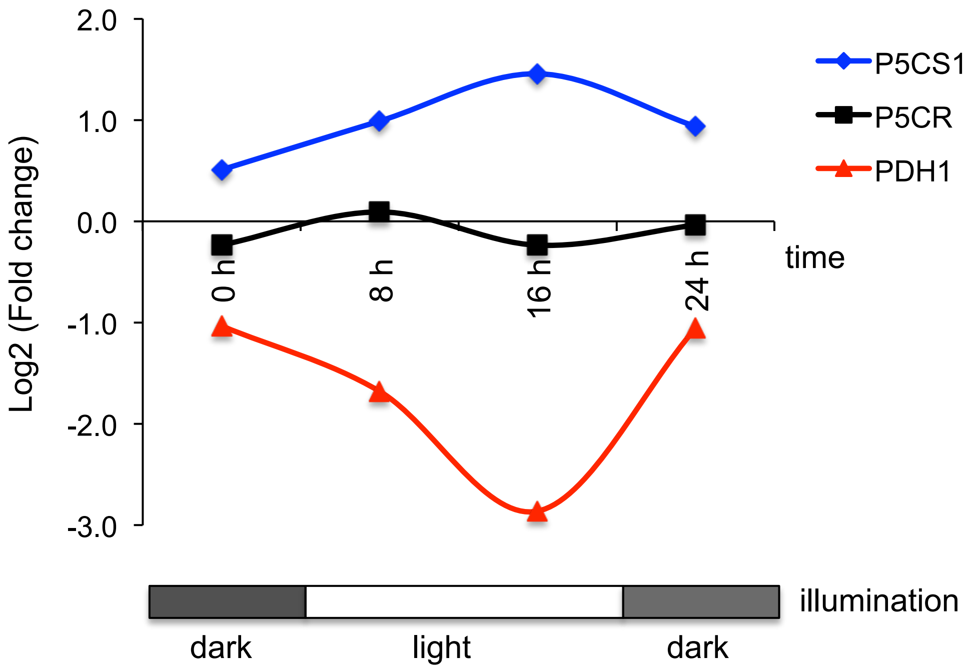

Data were compiled from supplementary files of transcript profiling data. Indicated values represent Log2(fold-change) of drought vs. well-watered conditions at each time point.

Reference: Dubois M, Claeys H, Van den Broeck L, Inze D (2017) Time of day determines Arabidopsis transcriptome and growth dynamics under mild drought. Plant Cell Environ **40:** 180-189.

Figure S2. Effect of externally added sucrose, glucose and mannitol on proline accumulation in dark-adapted plants.

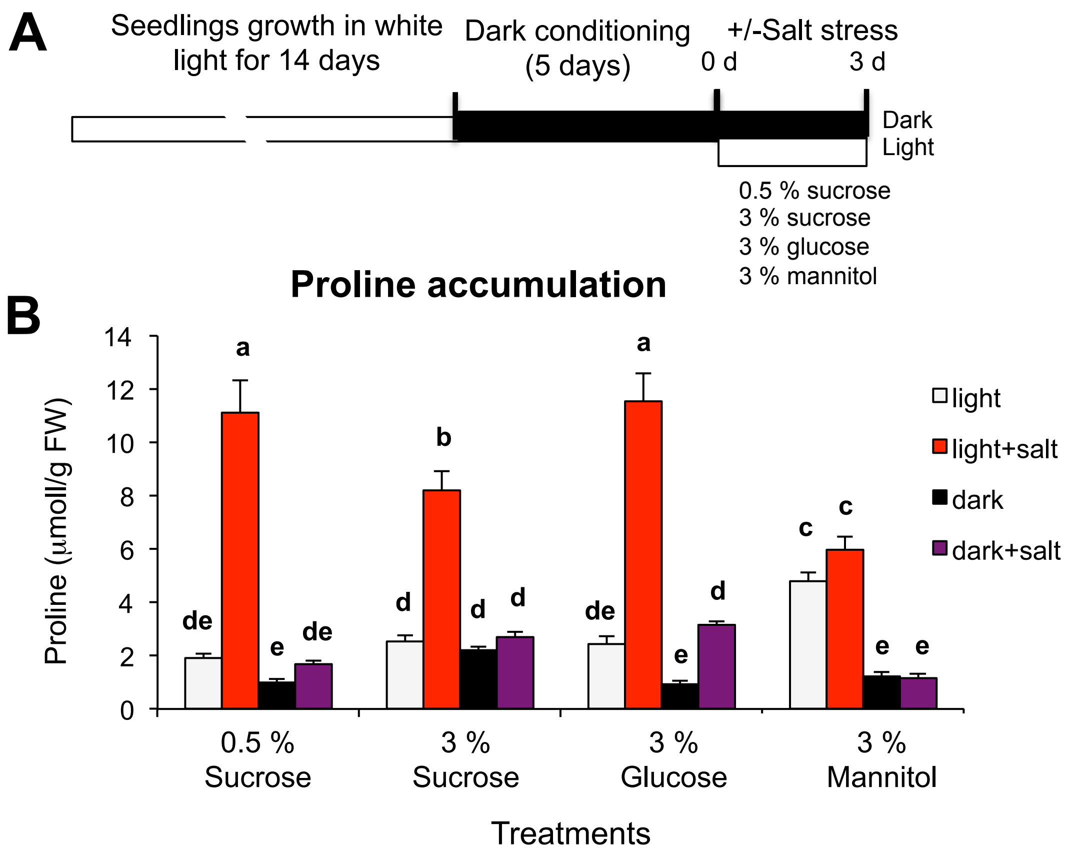

A) Experimental design. Fourteen-days-old wild type Arabidopsis plants were transferred to dark and after 5 days conditioning they were transferred to media containing 0.5 % sucrose (standard growth medium), 3 % of sucrose, 3 % glucose or 3 % mannitol, and treated with 150 mM NaCl under white light or kept in darkness. Concentrations are in W/V %. B) Proline accumulation in plants treated for three days. Note, that salt treatment enhanced proline levels in illuminated plants, but not or only minimally enhanced it in dark-treated plants. None of the sugars (sucrose, glucose) or sugar alcohol (mannitol) could promote proline accumulation in darkness. Error bars indicate standard deviation. Significant differences between means are shown by different letters ($p<0.05$, Two-way ANOVA, Duncan's test, N=5, fixed parameters were stress and sugar treatments).

Figure S3. Effect of different light regimes on proline accumulation in Arabidopsis Columbia (Col-0) and Wassilewskija (WS) ecotypes.

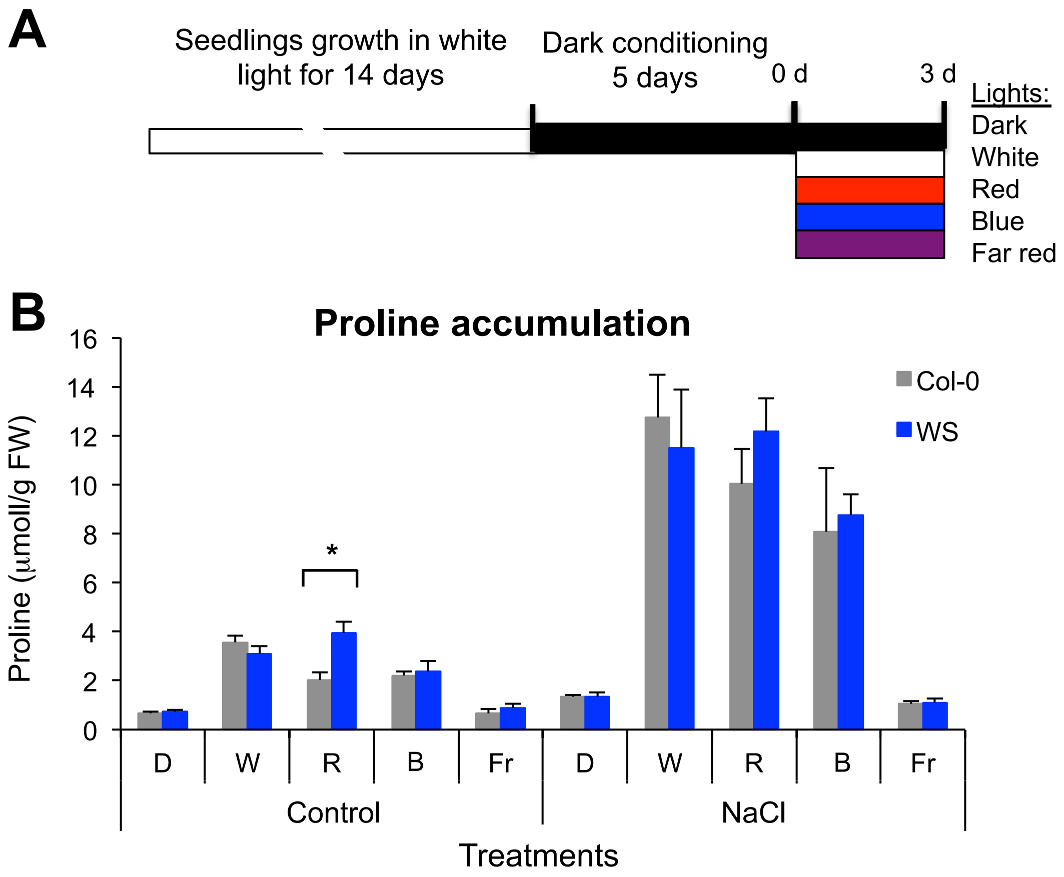

A) Experimental design. Dark-adapted Arabidopsis plants were transferred to white (W), red (R), blue (Bl), far red (Fr) lights or kept in dark (D) and treated with (NaCl) or without (Ctr) 150 mM NaCl. Free proline contents were determined in plants three days later. B) Proline levels in Col-0 and WS ecotypes. Note, that change in proline levels is similar in both Col-0 and WS ecotypes. Significant differences between Col-0 and WS are shown: * $p<0.05$, (Two-way ANOVA, Tukey test, N=5).

Figure S4. Effect of different light regimes on *P5CS1* and *PDH1* expression in Col-0 wild type plants.

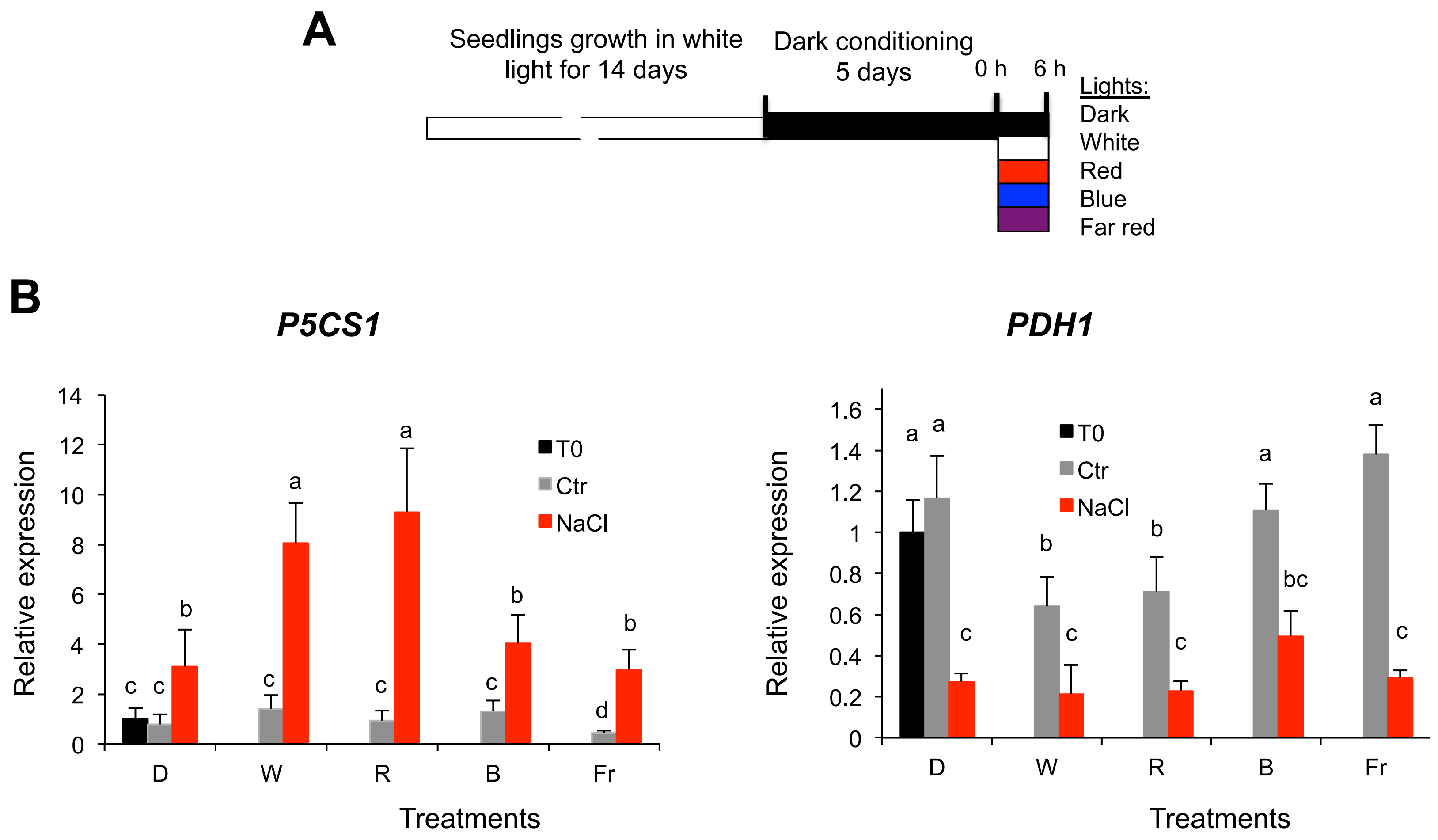

Results of a representative experiment. A) Experimental design. Dark-adapted Arabidopsis plants were transferred to white (W), red (R), blue (B) and far red (Fr) lights or kept in dark (D) and treated with or without 150 mM NaCl (NaCl or Ctr, respectively). RNA samples and cDNA templates were prepared in three repetitions. Gene expression of *P5CS1* and *PDH1* were determined in whole plants after 6 hours using quantitative RT-PCR (reference: *Actin2*). Relative transcript levels are shown as $2^{-\Delta\Delta Ct}$, where transcript levels in dark samples corresponds to 1. Error bars indicate standard deviation (N=3). Significant differences between means are shown by different letters ($p<0.05$, One-way ANOVA, Tukey test).

Figure S5. Effect of DCMU on proline metabolism in Arabidopsis.

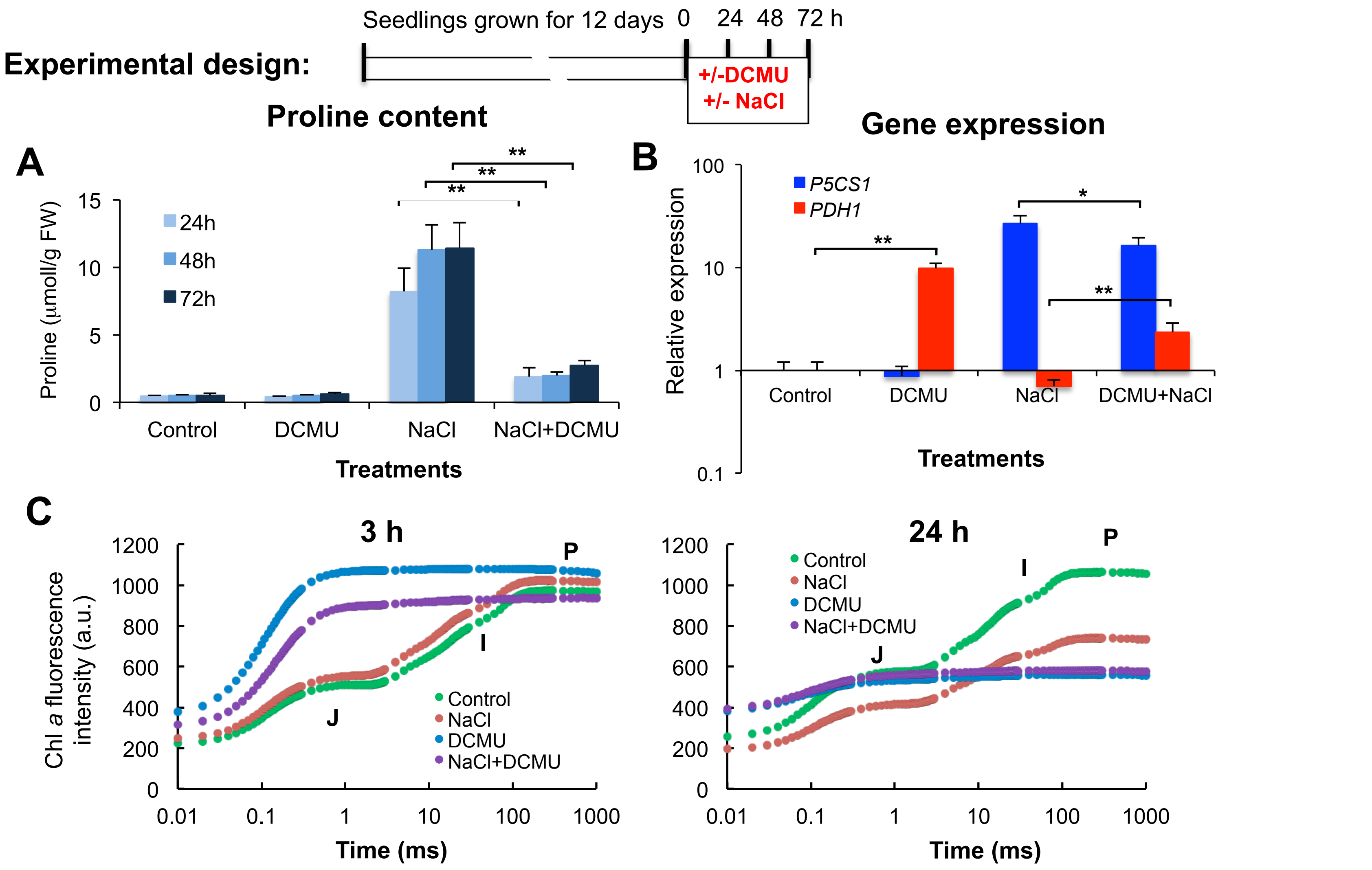

Fourteen-day-old *in vitro*-grown plants were treated with 150 mM NaCl in the presence or absence of 50 µM DCMU for up to 72 hours. A) Proline accumulation in DCMU and salt-treated plants. Columns represent the mean of N=5 samples, error bars indicate standard deviation. B) Expression of *P5CS1* and *PDH1* genes after 24 hours of treatments. Relative expression is shown on a logarithmic scale, where 1 equals transcript levels of non-treated plants. Error bars represent standard deviation of three repetitions. Significant differences between DCMU treated and not-treated values are shown by asterisk (*P<0.05, **P<0.01, One-way ANOVA, Tukey test). C) Chlorophyll *a* (OJIP) transients of stressed and control plants 3 hours and 24 hours after treatments. Experiments were repeated twice with similar results.

Figure S6. ChIP-seq result of HY5 binding around the *P5CS1* locus.

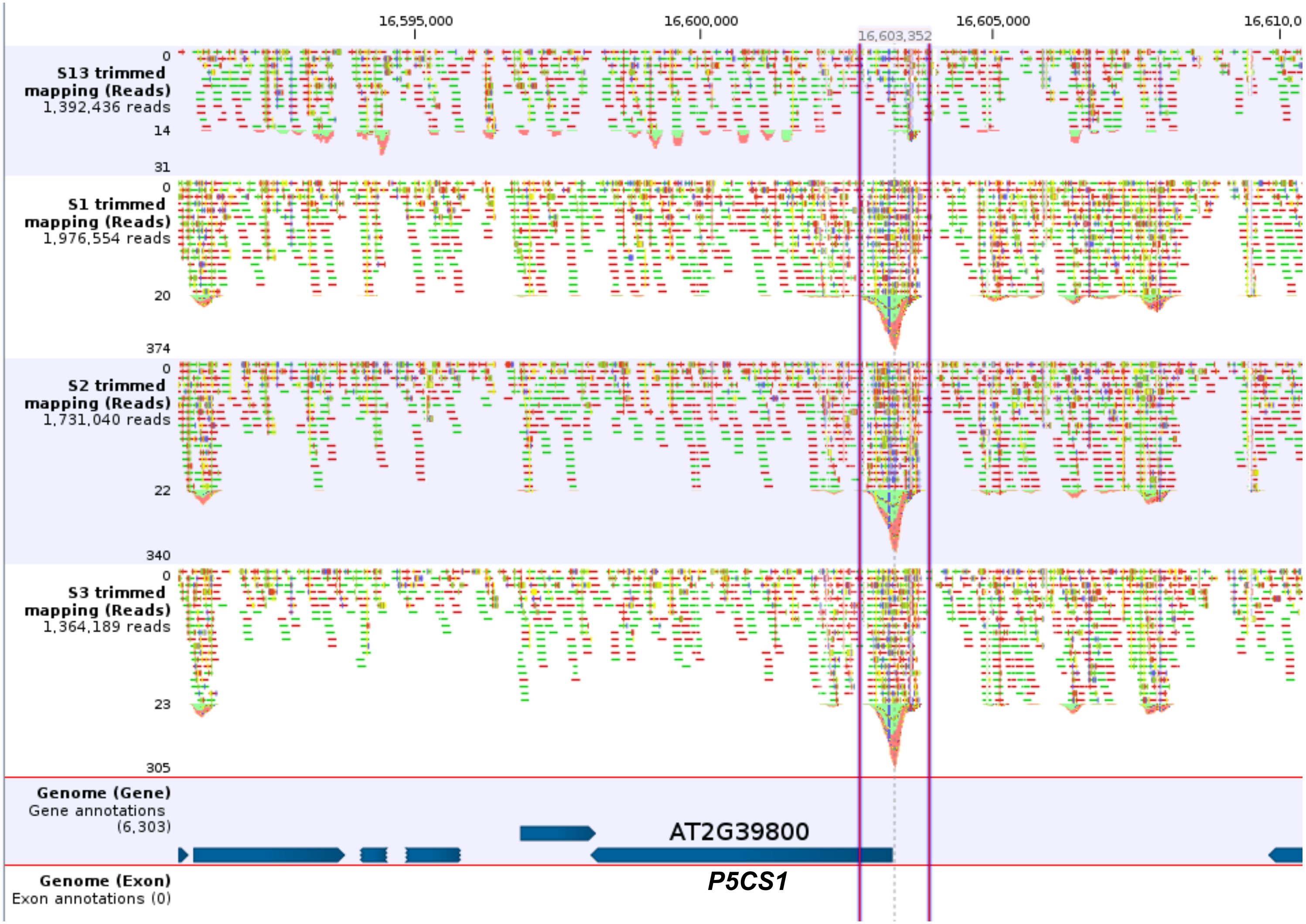

Data were compiled from ChIP-seq experiment published in Hajdu et al., (2018) Plant J **96:** 1242-1254. Reads from the non-immunoprecipitated mock control (S13) and from three independent biological samples (S1, S2 and S3) were mapped against the Arabidopsis genome (TAIR10). Blue arrow indicates the 5.2 kb transcribed region of *P5CS1*. Vertical pink lines indicate the approximate borders of read enhancements. Note that peak of the reads is close to the transcription initiation site of *P5CS1*. More experimental details and the complete set of raw data can be found at the NCBI GEO repository under the accession number GSE117797 (https://www.ncbi.nlm.nih.gov/geo/query/acc.cgi?acc=GSE117797).

Figure S7. ChIP-seq result of HY5 binding around the *PDH1* locus.

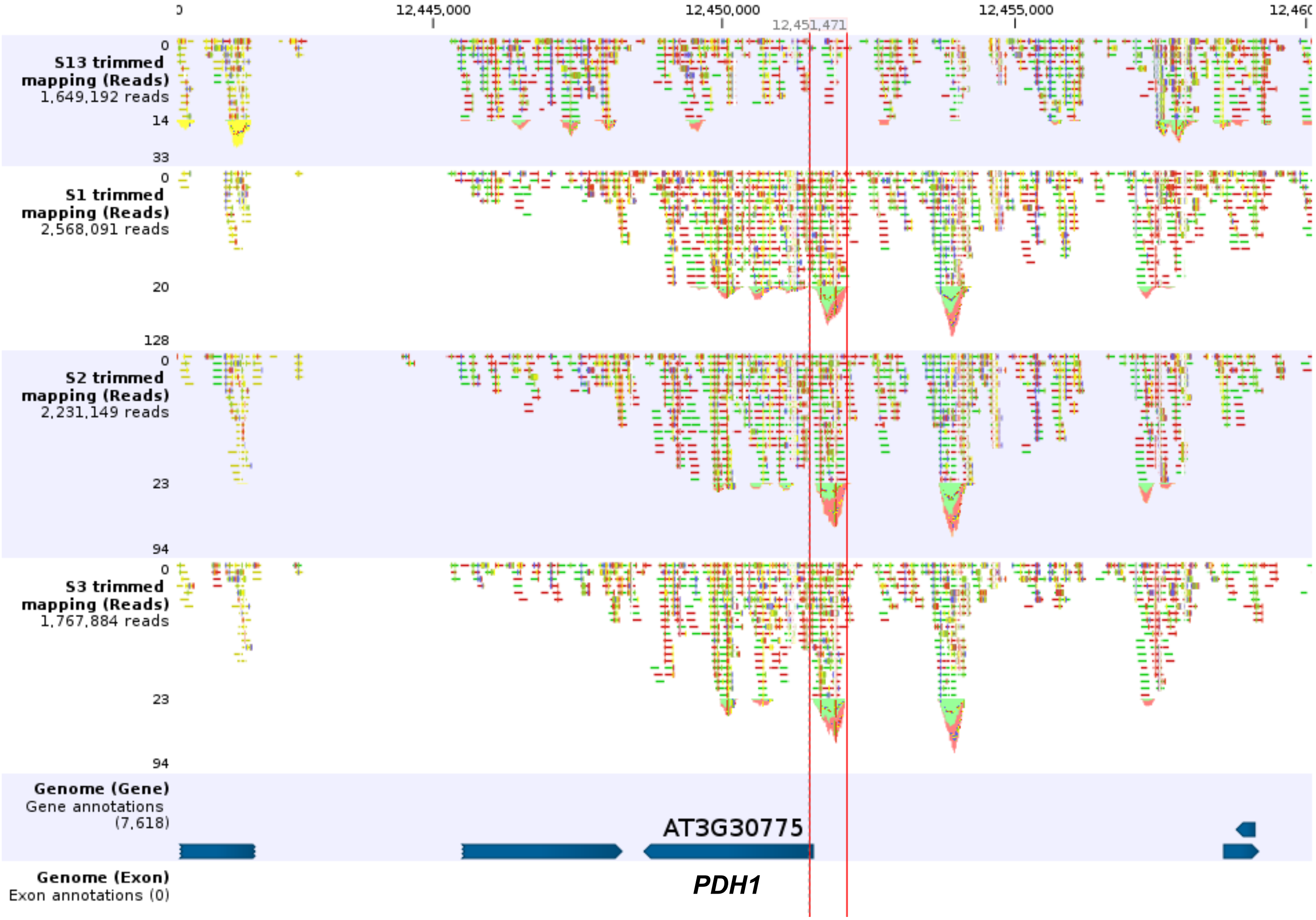

Data were compiled from ChIP-seq experiment published in Hajdu et al., (2018) Plant J **96:** 1242-1254. Reads from the non-immunoprecipitated mock control (S13) and from three independent biological samples (S1, S2 and S3) were mapped against the Arabidopsis genome (TAIR10). Blue arrow indicates the 2.9 kb transcribed region of *PDH1*. Vertical red lines indicate the approximate borders of read enhancements. Note that peak of the reads is approximately in 0.5 kb distance from the transcription initiation site of *PDH1*. More experimental details and the complete set of raw data can be found at the NCBI GEO repository under the accession number GSE117797 (https://www.ncbi.nlm.nih.gov/geo/query/acc.cgi?acc=GSE117797).

Figure S8. Proline accumulation in wild type Arabidopsis (WS) and in the *hy5hyh* mutant.

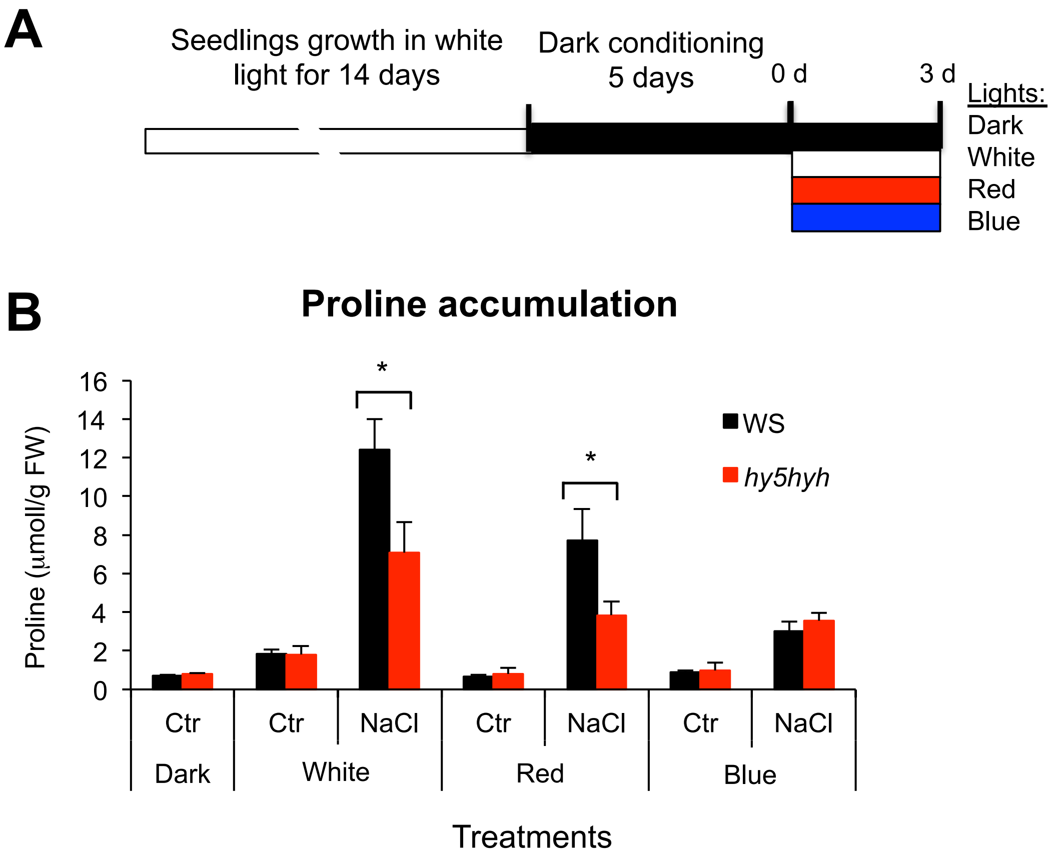

Proline content was determined in WS wild type and hy5hyh mutant plants. A) Experimental design. Dark-adapted Arabidopsis plants were transferred to white (W), red (R), blue (B) lights or kept in dark (D) and treated with (NaCl) or without (Ctr) 150 mM NaCl. Free proline contents were determined in whole plants three days later. B) Proline levels in WS ecotype and in *hy5hyh* mutant. Significant differences between WS and *hy5hyh* are indicated as: * $p<0.05$, (Two-way ANOVA, Tukey test, fixed parameters were genotypes and treatments, N=8).

Figure S9. Expression of *P5CS1* and *PDH1* genes in wild type Arabidopsis (WS) and in the *hy5hyh* mutant

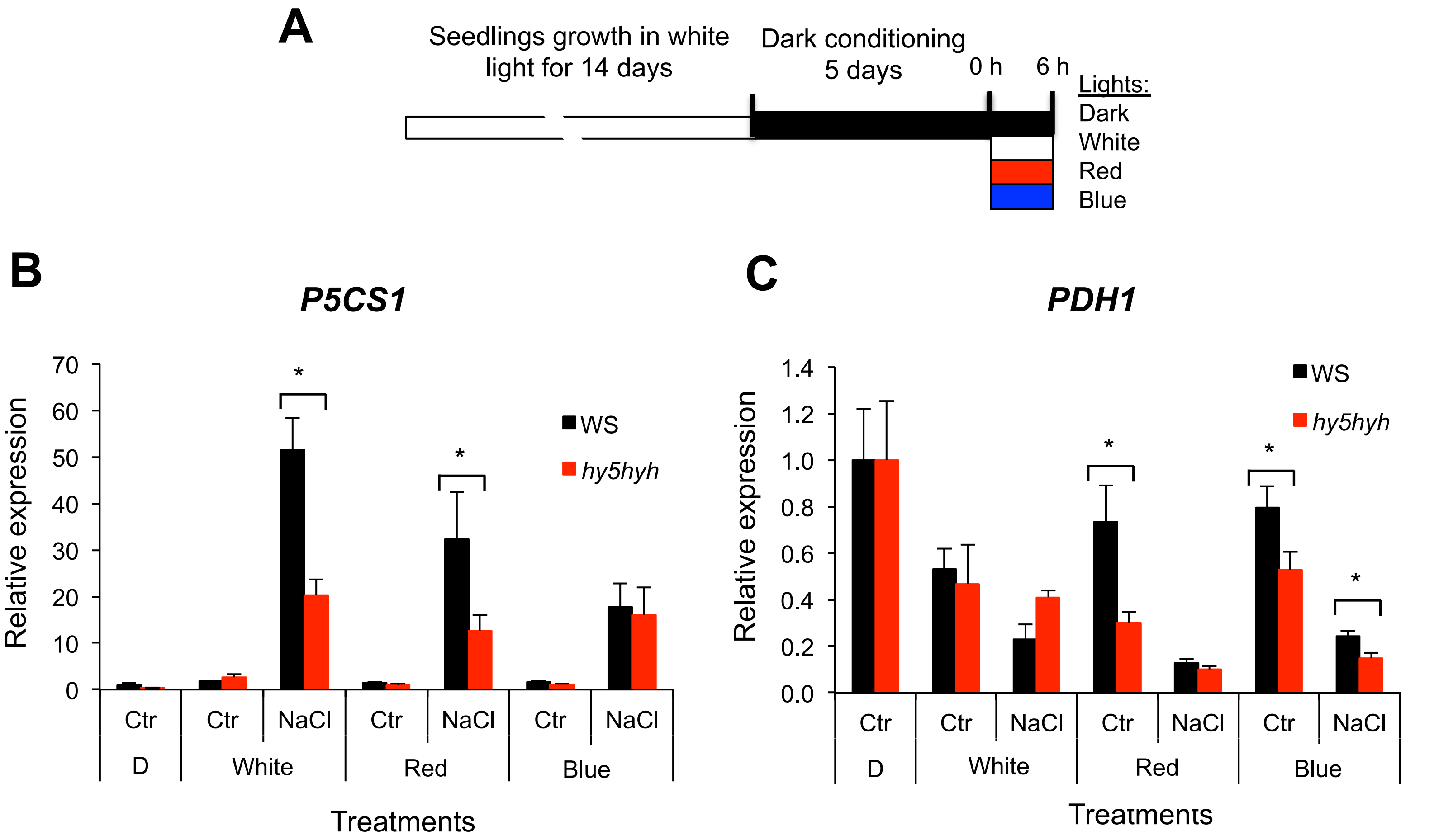

Results of a representative experiment A) Experimental design. Dark-adapted Arabidopsis plants were transferred to white (W), red (R), blue (B) lights or kept in dark (D) and treated with (NaCl) or without (Ctr) 150 mM NaCl. B,C) Expression of *P5CS1* (B) and *PDH1* (C) were determined in whole plants after 6 hours using quantitative RT-PCR (reference: Actin2). Relative transcript levels in WS ecotype and in *hy5hyh* mutant are shown as $2^{-\Delta\Delta Ct}$, where transcript levels in dark samples corresponds to 1. Significant differences between WS and *hy5hyh* are indicated as: * $p<0.05$, (Two-way ANOVA, Tukey test, fixed parameters were genotypes and treatments, N=3).
